# The Impact of Sociodemographic Disparities on Health-Related Quality of Life for Adults with Gliomas

**DOI:** 10.3390/cancers18050718

**Published:** 2026-02-24

**Authors:** Michael A. Perez, Devon C. Riegel, Joshua Amit Budhu

**Affiliations:** 1 Department of Neurology, Memorial Sloan Kettering Cancer Center, New York, NY 10065, USA; 2 Department of Neurology, Weill Cornell Medicine, New York, NY 10065, USA

**Keywords:** health-related quality of life, quality of life, healthcare disparities, neuro-oncology, gliomas

## Abstract

Health-related quality of life is a multidimensional concept used to describe one’s well-being in relation to their disease. Individuals with gliomas, the most commonly occurring malignant primary brain tumor, experience a multitude of symptoms due to their disease and treatments. Using the World Health Organization’s health-related quality-of-life model, we review the impact of this disease across each domain of the model. Furthermore, we summarize known sociodemographic disparities that contribute to inequities in health-related quality of life and highlight areas that require further investigation. Finally, we discuss potential areas for innovation to better address current gaps and inequities affecting the health-related quality of life of individuals with gliomas.

## Simple Summary

This article reviews the impact of glioma diagnoses and treatments on patients’ quality of life. Because gliomas affect the brain, they can influence physical, cognitive, emotional, and social functioning in ways that extend beyond traditional clinical outcomes. Using the World Health Organization’s health-related quality-of-life framework, this review examines the multiple domains of life affected by gliomas and highlights opportunities to improve patient-centered outcomes through clinical care, care delivery, and broader health system and policy approaches.

## 1. Introduction

Gliomas are the most common malignant primary brain tumors, with an estimated average age-adjusted annual incidence of 6 cases per 100,000 individuals [1]. Gliomas are classified into oligodendrogliomas, astrocytomas, and glioblastomas, with glioblastoma representing the most common subtype and having the poorest prognosis [1]. Prognosis varies widely across gliomas and depends on tumor type, grade, and molecular markers [2]. Individuals with lower-grade gliomas and/or favorable tumor markers such as isocitrate dehydrogenase (IDH) mutations may survive for years or decades, while those with glioblastoma, a high-grade glioma (HGG) that is IDH-wildtype, have a median overall survival of 15 months despite standard of care therapy [2]. Health outcomes for individuals with gliomas are also influenced by social determinants of health (SDOH), including economic, structural, and social conditions that shape access to and quality of care, which contribute to observed health disparities [3,4]. For example, patients who are uninsured or identify as Black or Latino are less likely to receive gross total resection, chemotherapy, or radiation therapy, treatments that have been shown to prolong overall survival in gliomas [5,6].

Individuals with gliomas experience a variety of presenting symptoms that depend on tumor location, with most tumors arising in the frontal, parietal, or temporal lobe [2]. Common presentations include headaches from mass effect or increased intracranial pressure, seizures, and focal neurological deficits, such as language impairment, motor or sensory deficits, or difficulties with cognitive functioning [2]. While surgical resection and anti-neoplastic therapy may alleviate symptoms, many individuals experience persistent or chronic symptoms requiring ongoing supportive management. Despite best supportive practices, individuals with gliomas report significant impairments in quality of life across physical, cognitive, and psychosocial domains, irrespective of tumor [7].

Health-related quality of life (HRQoL) is a subjective, multidimensional concept that captures an individual’s perception of their physical, psychological, and social well-being in the context of disease [8]. While HRQoL was historically less emphasized in clinical trials, advances in measurement tools, the emergence of more effective treatments, and the transformation of many diseases into more manageable or chronic conditions have led to its increased and appropriate incorporation as a patient-centered endpoint for evaluating the net benefit of therapies [9,10]. In response, the World Health Organization (WHO) developed a conceptual framework to standardize the assessment of HRQoL across diseases and cultural contexts. Within the WHO model, core domains of HRQoL include physical health, level of independence, psychological well-being, social relationships, environmental factors, and spirituality, religion, or personal beliefs (Table 1). Accordingly, this review applies the WHO HRQoL framework to examine quality of life and health disparities among individuals with gliomas.

## 2. Health-Related Quality of Life Among Individuals with Gliomas

### 2.1. Physical Capacity

Physical capacity is a core domain of HRQoL, reflecting an individual’s ability to engage in daily activities, which may be limited by pain, discomfort, fatigue, or impaired rest related to disease or its treatment [11]. More than 50% of individuals with gliomas experience pain or discomfort, most commonly headaches related to increased intracranial pressure or mass effect. These headaches are routinely managed with surgical resection or medications to reduce peri-tumoral edema, such as steroids or bevacizumab, a monoclonal antibody targeting vascular endothelial growth factor (VEGF). In other areas of neuro-oncology, studies have documented disparities in the recognition and management of symptom burden. For example, research shows that among individuals with brain metastases, racial and socioeconomic factors influence the use of supportive medications, including analgesics for headache [12]. However, it remains unclear whether similar disparities exist in the recognition and management of symptoms among individuals with gliomas. Notably, evidence suggests that Latino individuals are more likely to report unmanaged pain and sensory symptoms [13].

Studies have consistently documented racial and ethnic differences in pain management in other cancer populations [14]. Black patients are less likely to be prescribed opioids or referred to palliative care or hospice services and are more likely to report poorer pain control and lower HRQoL [15]. Within glioma populations, prior research has demonstrated racial, ethnic, age, and socioeconomic disparities in access to treatment, including differences in receipt of surgery, radiation therapy, and chemotherapy, suggesting that structural inequities in care delivery may also extend to supportive and symptom-directed [6,16,17]. Although disparities in pain management have not yet been consistently demonstrated among individuals with gliomas, these findings underscore the importance of standardized, proactive approaches to symptom management. One study demonstrates disparities in hospice enrollment, with non-White patients, individuals living in rural areas, men, and those with lower educational attainment experiencing lower hospice utilization [18]. Accordingly, we recommend routine consideration of palliative care referral for all patients with uncontrolled pain, as a strategy to improve symptom control and reduce the risk of inequities in care.

Fatigue, defined as tiredness, weakness, and lack of energy, is widely prevalent among individuals with gliomas, with estimates ranging from 20 to 76% [19,20]. The etiology of fatigue in this population is considered multifactorial, with contributing factors including tumor-related characteristics, comorbid conditions, psychological symptoms, and treatment-related side effects [21]. Contrary to common assumptions, studies evaluating tumor type and treatment modalities have not consistently identified differences in the prevalence or severity of fatigue [22]. These findings are corroborated across other cancer types and their related treatments [23]. Available evidence suggests that socioeconomic disadvantage, rather than tumor- or treatment-related factors, may be associated with worse patient-reported fatigue in individuals with glioma [24].

Studies evaluating associations between fatigue and clinical factors such as tumor location, use of antiepileptic drugs, and sex have yielded conflicting results [19,23,25]. While sedation is a well-known side effect of antiepileptic drugs, small studies evaluating fatigue among individuals with gliomas have yet to unequivocally establish an association between antiepileptic drugs and fatigue [19,23]. In contrast, psychosocial and functional factors, including depression, baseline preoperative fatigue, and physical functioning, have been more consistently identified as predictors of postoperative fatigue [23]. One proposed mechanism for fatigue in neurologic conditions is the increased cognitive or physical effort required to accomplish everyday tasks [26]. However, adults with gliomas appear to experience fatigue independently of neurocognitive impairment [27].

Efforts to mitigate fatigue among individuals with gliomas have had varying success. The National Comprehensive Cancer Network (NCCN) guidelines recommend evaluating for treatable contributing factors such as pain, emotional distress, physical deconditioning, sleep disturbances, and medication side effects [21]. After addressing contributing factors, both nonpharmacologic and pharmacologic interventions may be considered [21]. Regular physical activity is one nonpharmacologic strategy that has been associated with reductions in fatigue among individuals with gliomas [28,29]. However, disparities in the built environment may limit the feasibility of exercise for individuals from lower socioeconomic or marginalized communities, including reduced access to parks, green spaces, and fitness facilities, as well as financial barriers associated with gym membership and safe recreational spaces [30,31]. Meanwhile, pharmacologic interventions such as the use of armodafinil, a psychostimulant, have not been found to provide a meaningful benefit [32]. Similarly, trials of methylphenidate in individuals with ADHD have not shown consistent efficacy [33]. However, the NCCN recommendation for the management of fatigue is to trial a psychostimulant after addressing other contributing factors [21].

### 2.2. Psychological

Psychological factors influencing HRQoL include mood, self-perception, and cognition. A chief concern among individuals with gliomas is neurocognitive dysfunction, which commonly manifests as word-finding difficulties, short-term memory impairment, and difficulty completing complex tasks [34]. While radiation therapy can contribute to the development of cognitive changes, studies reveal that individuals with gliomas experience neurocognitive dysfunction independently of radiation exposure [35]. In one study using standardized neuropsychological testing, 74% of individuals with gliomas exhibited clinically significant impairment in at least one cognitive domain [34]. A meta-analysis evaluating cognitive impairment among individuals with gliomas revealed greater rates of cognitive impairment for high-grade gliomas compared to low-grade gliomas, affecting 68.9% and 31.1% of individuals, respectively [36]. Evaluation of sociodemographic factors suggests an association between cognitive impairment and lower educational achievement and non-White racial background [37]. However, more in-depth analyses of cognitive dysfunction are often limited by a heterogeneity of cognitive testing and a lack of sociodemographic diversity among participants.

Another common detriment to HRQoL for individuals with gliomas is mood disturbance. Studies indicate that 38–48% of individuals experience clinically significant symptoms of anxiety and/or depression during their disease [38,39]. These psychological stressors may be further influenced by treatment-related changes in physical appearance such as steroid-related weight changes, alopecia, and surgical scars. While sufficient data are not available to evaluate for racial or ethnic disparities among individuals with gliomas and depression, higher rates of depression have been observed among Black individuals with other cancers [40,41]. Additionally, Black individuals are less likely to be prescribed antidepressants [40]. Research demonstrates high rates of depressive symptoms in individuals with gliomas and identifies socioeconomic factors, including lower income and related sociodemographic variables, as risk factors for depression in this population. For example, a cross-sectional study of individuals with glioma found that lower household income, marital status, and sleep disturbances were independently associated with depression symptoms [42]. Furthermore, analyses of socioeconomic deprivation in primary malignant brain tumors show that greater deprivation is associated with worse emotional distress, including depression [24]. These findings parallel the broader literature on cancer survivorship, showing that social determinants and healthcare access disparities can affect mental health outcomes [43,44,45].

Other factors associated with mood disturbances in individuals with gliomas include female sex and a history of psychiatric illness [39,46]. Consistent with neuroanatomic vulnerability, individuals with gliomas involving their limbic system experience higher rates of severe depressive symptoms [47]. Evaluation of depression, fatigue, emotional distress, and existential problems among individuals with gliomas revealed that depressive symptoms emerged as the strongest independent predictor of HRQoL [38]. Despite the strong relationship between mood disturbances and HRQoL, there is no interrelationship between mood disorders and neurocognitive symptoms [48]. This dissociation emphasizes the importance of independent screening of psychological symptoms and cognitive impairment in both clinical care and clinical trials, rather than assuming overlap between these domains [48].

### 2.3. Level of Independence

Level of independence refers to an individual’s ability to perform activities of daily living (ADLs) and instrumental activities of daily living (IADLs) [49]. ADLs and IADLs include self-care, grooming, financial management, medication management, and employment. Given the central importance of maintaining independence, several functional measures, most notably the Karnofsky Performance Scale (KPS), were developed to assess functional status longitudinally in individuals with brain tumors [50,51]. Importantly, significant inter-rater variability exists among clinicians when measuring KPS among individuals with brain tumors [52]. Emerging evidence suggests that clinician-assigned performance status measures, including the Karnofsky Performance Scale (KPS), may be subject to bias, with Black individuals more likely to be rated as having lower performance status [53]. Such differences may influence treatment recommendations and eligibility for clinical trials, potentially contributing to the lack of diversity observed in glioma clinical [54]. In response to concerns about bias in clinical assessment and decision-making, the Society for Neuro-Oncology has emphasized the importance of bias-mitigation strategies, including the use of “nudges”—behavioral-economics-informed interventions designed to promote more equitable and consistent clinical decision-making [55].

Despite variability in KPS measurement, multiple studies have consistently found that functional independence declines with each tumor [56,57]. Consequently, innovations in glioma treatment have increasingly focused on delaying disease progression and preserving or restoring independence at tumor recurrence. An example of success in surgical innovation to preserve independence is the use of awake craniotomies to safely resect tumors near eloquent cortex [58]. Similarly, over half of individuals receiving chemotherapy for treatment of glioma recurrence experience stability or improvement in several measures of HRQOL, including physical function [59]. Unfortunately, significant disparities in access to high-volume medical centers with expertise in awake craniotomies and brain tumor care exist [60]. Individuals who identify as Latino, are uninsured, or come from a low-income household are less likely to receive care at a high-volume medical center [60], raising concern for disparities in functional outcomes related to these sociodemographic factors.

Employability represents another key metric of independence and is substantially affected among individuals diagnosed with gliomas. Return-to-work rates vary by tumor grade, with approximately half of individuals with low-grade gliomas returning to work after diagnosis compared to one-third of individuals with high-grade [61,62]. To date, limited data exist evaluating racial or ethnic disparities in return-to-work rates among individuals with gliomas. However, studies in other cancer populations have found that Black and Latino patients are less often able to return to work after a cancer diagnosis [63,64]. Additionally, a European population-based study found that individuals from lower socioeconomic backgrounds experience lower overall survival compared to individuals from higher socioeconomic backgrounds [65]. Although the mechanisms underlying this association are multifactorial, in the United States, the link between employment and health insurance may further amplify these disparities [66]. Therefore, employability serves a key role not only in HRQoL but also in associated health outcomes. The phenomenon of “job lock,” whereby patients or caregivers remain employed to maintain health insurance coverage, may exacerbate psychosocial stress and financial toxicity, particularly in the context of serious illness [67,68].

A greater extent of tumor resection predicts successful return to work [69]. Meanwhile, the development of acute postoperative motor, gait, or cognitive deficits reduce the likelihood of returning to work [62]. Even when individuals with low-grade gliomas return to work, many experience ongoing occupational difficulties related to their illness [61]. Limitations include cognitive difficulties such as concentration and speed of task completion [61]. These findings underscore the need for further innovation in glioma treatments and cognitive rehabilitation to help individuals with gliomas regain their independence and experience greater HRQoL.

### 2.4. Social Relationships

The ability to maintain and nourish social relationships is another key metric of HRQoL. Individuals with gliomas are particularly susceptible to disruptions in social relationships, given the cognitive, language, and communication impairments that may result from their disease or related treatments [70]. Poor social relationships and social isolation have been associated with worsening neurologic illness and functional decline, underscoring their importance in neurologic health more broadly [70,71]. Quantitative assessments of social networks and social isolation for individuals with gliomas remain limited, although recent pilot studies have begun to explore social network structure in this population [70]. In a small study of nine individuals with glioblastoma, 77% reported dissatisfaction with personal relationships. Similarly, a qualitative study exploring the challenges faced by individuals with glioblastoma identified social isolation as a prominent subtheme, with participants describing difficulty engaging in conversations due to cognitive or language changes, followed by progressive withdrawal from social circles and community life [72].

While research regarding racial disparities in social relationships for individuals with gliomas has yet to be performed, racial disparities have been observed among individuals with breast cancers. Notably, social network size, network density, and practical support have been associated with increased 5-year survival among individuals with breast cancers [73]. However, Black individuals with breast cancers were found to have smaller networks, lower network density, and greater geographical separation from support compared to their White counterparts [73]. Similar patterns have been observed in other areas of medicine; for example, strong social networks were identified as a protective factor during the COVID-19 pandemic, whereas limited access to social support among older adults and marginalized populations was thought to contribute, in part, to observed disparities in outcomes [74]. Given the substantial caregiving needs and social challenges faced by individuals with gliomas, further investigation into disparities in social networks would be helpful to inform future interventions.

Another important dimension of social relationships is sexual health. Sexual health is associated with better overall health, well-being, and enjoyment of life [75,76,77]. Meanwhile, sexual dysfunction can negatively affect interpersonal relationships and psychosocial functioning [77,78]. Among individuals with low-grade gliomas who are not receiving adjuvant therapy, 44–63% report sexual dysfunction, most commonly related to sexual drive and satisfaction [77,79,80]. As a consequence of decreased sexual arousal, sexual desire, and orgasms, women report lower levels of social well-being and quality of relationships [77,80]. Despite the high prevalence of sexual health concerns, screening and discussion of sexual dysfunction remain uncommon in neuro-oncology care, with 59% of neurosurgeons reporting never having discussed sexual health with their patients [81]. Routine assessment of sexual health should be performed among individuals with gliomas, particularly given the availability of effective interventions targeting sexual desire and satisfaction, including pharmacologic therapies and sexual aids [82,83].

### 2.5. Environment

Environmental factors that influence one’s HRQoL include financial stability, physical safety, transportation, access to healthcare, and the ability to engage in leisurely activities. For individuals diagnosed with gliomas, financial toxicity represents a major environmental determinant of diminished quality of life. Financial toxicity encompasses the negative impact and economic burden of medical care, including but not limited to medical debt, loss of income, and the associated psychological distress [84,85]. Individuals with cancer often incur high out-of-pocket costs, many of which are not fully covered by insurance [85]. For example, one study found that 34% of individuals with glioblastoma and 54% of their caregivers spent more than $271 USD out-of-pocket per month on treatment [86]. A separate analysis reported that the mean per-patient per-month cost for individual with glioblastoma was $18,053 USD when including both in- and outpatient costs [87,88]. Even when patients have insurance coverage, co-pays for treatment can be substantial, ranging from $0–100 USD to >$1000 USD per chemotherapy prescription [89]. As neuro-oncology treatment paradigms evolve, including the introduction of targeted therapies such as isocitrate dehydrogenase (IDH) inhibitors, overall treatment costs may increase and be partially passed on to patients. With total annual costs that can exceed $300,000 USD, a portion of these expenses may be borne directly by patients through medication co-payments or indirectly through the healthcare system, potentially contributing to higher insurance premiums.

**Figure 1:**
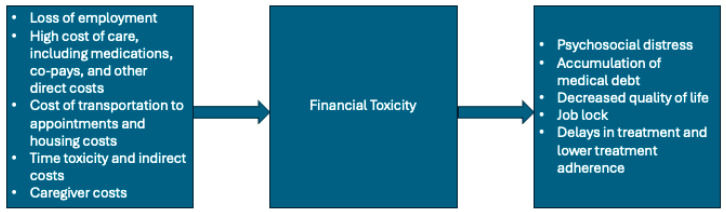
This figure summarizes the factors contributing to the development of financial toxicity and the repercussions of financial toxicity.

As a consequence of these cumulative costs, patients may acquire high medical debt across multiple points of care, including outpatient visits, which can force trade-offs between medical expenses and essential needs such as housing, food, and transportation [85,90]. Compounding these economic toxicities, individuals with gliomas face a high risk of unemployment or reduced work capacity due to disease-related disability, further limiting financial stability [84,85]. Additionally, these patients are at a high risk of transportation insecurity, which has been associated with missed appointments, treatment delays, and foregone cancer care, thereby exacerbating treatment inequities and ultimately overall survival [65,91]. Ultimately, limited financial resources can profoundly impair QoL and contribute to inequities in cancer treatment and outcomes (Figure 1).

Another environmental challenge experienced by individuals with gliomas is the ability to participate in recreation and leisure activities, which may be limited by neurocognitive impairments related to disease or treatment. In addition to cognitive dysfunction, neurologic symptoms, including motor and sensory deficits, aphasia, seizures, and headaches, can interfere with occupational and leisure activities [85,92,93]. Low-grade gliomas are more likely to be epileptogenic, and as a result, individuals with gliomas are more likely to avoid activities that they previously enjoyed because of seizure risk [92,94]. One study found that nearly half (48%) of all individuals with gliomas surveyed reported moderate–severe activity-related interference, while another found that poor physical health among individuals with glioblastoma led to fewer outings, and, in turn, lower subjective well-being [56,95]. In sum, these findings highlight substantial barriers to participation in leisure activities and skill acquisition, which negatively impact overall HRQoL among individuals with glioma.

Sociodemographic factors further shape environmental influences on QoL. Individuals with gliomas who grew up in or currently reside in rural areas are more likely to experience moderate to severe activity interference related to their disease [56]. These individuals also tend to travel longer distances to receive care, resulting in significant time toxicity, defined as the time spent receiving medical care that displaces time otherwise available for work, caregiving, or leisure activities [91,96,97]. In urban settings, individuals from racial and ethnic minority groups, those with lower median household income, and individuals with public insurance are more likely to seek care through emergency departments, sometimes presenting with more advanced disease, due to barriers to continuity of outpatient care [98]. Addressing these environmental and structural disparities in care will require large-scale, system-level interventions to improve equitable access to care, treatment outcomes, and HRQoL [4].

### 2.6. Spirituality

Spirituality is defined as a personal quest for meaning and understanding in relation to existential questions about life and one’s connections to the sacred or transcendent [99,100]. While it is related to religiosity, spirituality is distinct in that religiosity emphasizes organized practices, beliefs, and worship [100]. Spirituality has been associated with more effective coping with chronic and serious illness, as well as improved cancer-related symptom control [101,102,103]. These associations are thought to underlie the positive relationship between spirituality and HRQOL [100]. Racial and ethnic differences in spirituality have been described among survivors of other cancers, with individuals who identify as Black or Latino reporting higher levels of meaning and peace, contributing to greater spiritual well-being [104]. Among individuals with gliomas, similar findings have been observed, with studies demonstrating a positive association between spirituality or religiosity and higher HRQoL, independent of performance status or tumor type [105]. Furthermore, individuals with glioblastoma who report higher spiritual well-being have also demonstrated greater resilience and longer survival [106]. Given the shortened life expectancy associated among individuals with gliomas, these findings emphasize the importance of acknowledging and supporting spirituality as part of comprehensive, patient-centered care throughout the disease course [105].

## 3. Discussion

**Figure 2:**
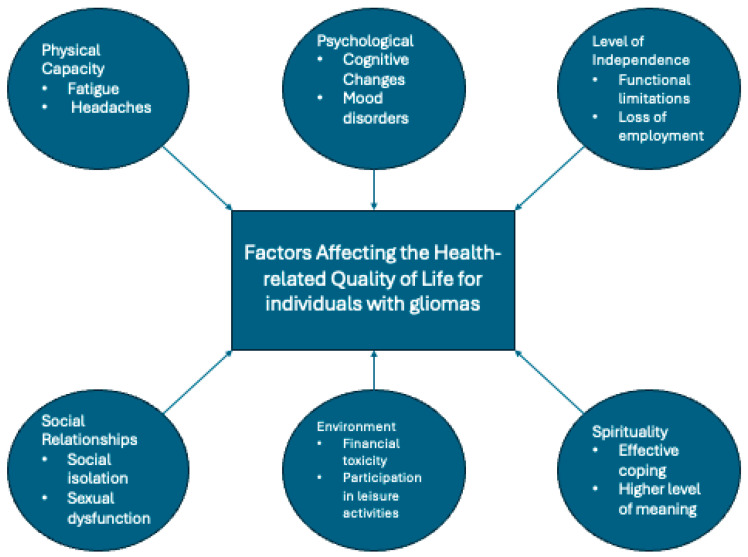
A summary of the factors affecting the HRQoL among individuals with gliomas.

Gliomas affect individuals across all domains of HRQoL (Figure 2). The degree and nature of impact vary according to tumor grade, tumor location, and sociodemographic factors, with cognitive impairment, social isolation, financial toxicity, mood disturbances, and loss of independence emerging as key contributors to diminished HRQoL. Although these challenges are not unique to individuals with gliomas, addressing them requires targeted, equity-conscious interventions that account for the heterogeneity of patient experiences and the social determinants of health.

Several pragmatic opportunities exist to improve HRQoL in neuro-oncology care. Standardized, universal screening for common and impactful concerns, such as mood disturbances, financial hardship, and sexual health, followed by timely referral to appropriate specialists, may substantially improve patient well-being. Partnerships with physiatrists and physical therapy specialists also offer important opportunities to develop tailored rehabilitation programs to help restore and maintain independence and support patients’ individual goals. Additionally, expanded use of virtual care may improve access to specialized brain tumor centers for patients who face geographic, transportation, or mobility barriers.

Clinical trials and research represent another critical avenue for advancing understanding of HRQoL. Recent clinical trials, including those evaluating vorasidenib, have increasingly incorporated HRQoL and patient-reported outcomes as key outcomes alongside traditional clinical endpoints [107]. The European Organization for Research and Treatment of Cancer (EORTC) developed a validated HRQoL questionnaire specifically designed to assess the impact of tumors and treatments on patients’ well-being. Broader adoption of standardized instruments can facilitate cross-trial comparisons, inform clinical decision-making, and guide health policy [108]. Additionally, as clinical trials have historically lacked sociodemographic diversity, new guidance has emerged to improve recruitment of diverse participants [55]. Expanding participant diversity and routinely integrating HRQoL assessments into glioma trials will be essential to better characterize disparities and inform more equitable care.

Beyond traditional efficacy trials, pragmatic clinical trials and quality improvement initiatives will be essential for advancing HRQoL in neuro-oncology. Pragmatic trials are designed to evaluate interventions under real-world clinical conditions, emphasizing usual care settings, broad patient inclusion, and outcomes that are directly relevant to patients and health systems [109]. Such approaches allow evaluation of real-world interventions such as routine HRQoL screening, integrated supportive care, rehabilitation, and financial navigation within diverse care settings. Prioritizing pragmatic and implementation-focused research alongside randomized trials will be critical to translating therapeutic advances into meaningful improvements in HRQoL for individuals with gliomas and should be a central focus of the field moving forward.

Importantly, there are currently no widely adopted guidelines for the standardization of HRQoL measurement instruments in glioma research, highlighting an important opportunity for professional societies and research consortia to collaborate on the development and implementation of standardized, patient-centered assessment tools. For all the reasons outlined in this review, it is our belief that an invigorated focus on improving the HRQoL of individuals with gliomas is critical to unlocking the full benefit provided by any current and future treatments.

## 4. Conclusions

Gliomas impose a substantial and multifaceted burden on HRQoL that extends well beyond survival, affecting physical functioning, cognition, emotional well-being, social relationships, independence, environmental stability, and spirituality. While advances in surgical, medical, and targeted therapies have improved disease control for some individuals, these gains are often accompanied by persistent symptoms, functional limitations, and structural barriers that disproportionately affect socially and economically marginalized populations.

Improving HRQoL for individuals with gliomas will require moving beyond disease-directed treatment alone. Standardized screening for common quality-of-life challenges, integration of supportive and rehabilitative services, and intentional efforts to address financial, geographic, and structural barriers to care represent actionable opportunities to improve patient-centered outcomes. At the research level, broader incorporation of HRQoL and patient-reported outcomes into clinical trials, greater sociodemographic representation, and the development of standardized assessment tools are critical next steps.

Finally, prioritizing pragmatic clinical trials and quality improvement initiatives will be essential to translating therapeutic advances into meaningful improvements in daily functioning and well-being for individuals living with gliomas. An intentional, equity-focused emphasis on HRQoL should be a central goal of neuro-oncology care and research to ensure that progress in treatment is matched by progress in patients’ lived experience.

## Figures and Tables

**Table 1:** A table summarizing the six domains of the World Health Organization’s (WHO) quality-of-life (QOL) model with contributing factors listed under each domain. This is an adaptation of the original work “WHOQOL User Manual.” Geneva: World Health Organization (WHO); 2012. License: CC BY-NC-SA 3.0 IGO.

Domain	Contributing Factors
Physical Capacity	Pain and discomfort
	Energy and fatigue
	Sleep and rest
Psychological	Positive feelings
	Thinking, memory, and concentration
	Self-esteem
	Bodily image and appearance
	Negative feelings
Level of Independence	Mobility
	Activities of daily living
	Dependence on medication or treatments
Social Relationships	Personal relationships
	Social support
	Sexual activity
Environment	Physical safety and security
	Home environment
	Financial resources
	Health and social care: accessibility and quality
	Participation in and opportunities for recreation/leisure activities
	Physical environment (pollution/noise/traffic/climate)
	Transportation
Spirituality/Religion/Personal Beliefs	

## Data Availability

Not applicable.
